# Supplementary material for: A time-dependent diffusion MRI signature of axon caliber variations and beading
Source: Commun Biol. 2020 Jul 7;3:354. doi: 10.1038/s42003-020-1050-x (PMC7341838; doi:10.1038/s42003-020-1050-x)
Supplement: Supplementary file 1 — Supplementary Information [file 42003_2020_1050_MOESM1_ESM.pdf]

A time-dependent diffusion MRI signature of axon caliber variations and beading Supplementary Information

Hong-Hsi Lee,* Antonios Papaioannou, Sung-Lyoung Kim, Dmitry S. Novikov, and Els Fieremans
Center for Biomedical Imaging and Center for Advanced Imaging Innovation and Research (CAI^{2}R), Department of Radiology, New York University School of Medicine, New York, NY 10016, USA

(Dated: May 29, 2020)

Supplementary Notes

Statistics of mitochondrial morphology. For segmented mitochondria in Fig. 6a, the mitochondrial surface area is 2.26 $\pm$ 2.11 μm^2, and the mitochondrial volume is 0.21 $\pm$ 0.25 μm^3. For an individual axon, the number of mitochondria per unit IAS volume is 0.32 $\pm$ 0.14 μm^{-3}, the ratio of mitochondrial surface area to IAS volume is 0.67 $\pm$ 0.32 μm^{-1}, and the volume fraction of mitochondria to IAS is 6.0 $\pm$ 3.0%, with histograms shown in Supplementary Fig. 1. All of these values are consistent with previous histological study in mouse optic nerve.[1]

Consequently, although the small mitochondrial volume ($\sim$6% of the IAS volume) suggests a relatively small effect on the dMRI signal, as shown in Fig. 2b-c, they indirectly may alter the diffusion time-dependence, since the mitochondrial distribution along axons correlates with axon caliber variation.

Human brain data of 10 additional subjects. The dMRI measurement was performed on 10 healthy subjects (7 males/3 females, 23-30 years old) by using a monopolar PGSE sequence provided by the vendor (Siemens WIP 511E) on a 3T Siemens Prisma scanner (Erlangen Germany) with a 64-channel head coil. For each subject, we varied diffusion time t = [21.2, 22, 24, 26, 28, 30, 40, 50, 75, 100] ms and fixed diffusion gradient pulse width δ at 15 ms. For each scan, we obtained one b = 0 non-diffusion weighted image and 64 DWIs of b-values b = [0.1, 0.4, 1, 1.5] ms/μm^2 along [4, 10, 20, 30] gradient directions for each b-shell, with an isotropic resolution (2 mm)^3 and a field-of-view (216 mm)^2. The scanned brain volume was a slab of 15 slices, aligned parallel to the anterior commissure to posterior commissure line. The CC was in the middle of the slab for covering the entire CC. All scans were performed with the same TR/TE = 5000/150 ms. Total acquisition time is $\sim$65 min for each subject.

Image processing pipeline and chosen ROIs are the same as the ones in the main text.

The time-dependent axial diffusivity $D(t)$, measured by monopolar PGSE in the human brain WM (Supplementary Fig. 2a-b), were averaged over 10 healthy subjects and plotted with respect to $1/\sqrt{t}$. In all WM ROIs except Midbody of CC, the axial diffusivity time-dependence demonstrates a $1/\sqrt{t}$ power-law relation in equation (1) (P-value $<$ 0.05, Supplementary Table 1), indicating that the universality class along WM axons is the short-range disorder (randomly distributed tissue inhomogeneity) in $1d$, corresponding to a dynamical exponent $\vartheta = 1/2$. The fitted parameters (c, D_∞) are shown in Supplementary Table 1.

Furthermore, the axial kurtosis in WM is $\sim$0.8 to 1, demonstrating the non-Gaussian diffusion along axons (Supplementary Fig. 2c-d).

The effect of axonal diameter distribution on axial diffusivity time-dependence. To investigate the effect of axonal diameter distribution on diffusivity time-dependence along axons, we performed Monte Carlo simulations in three different fiber bundles, composed of (1) realistic axonal shapes with caliber variations and axonal undulations (geometry I in Fig. 2a), (2) synthetic fibers with only axonal undulations (geometry IV in Fig. 2a), and (3) perfectly straight cylinders. The three fiber bundles have the same $2d$ cross-sectional diameter distribution and orientation dispersion based on Watson distribution. Simulation results in Supplementary Fig. 3 demonstrate that fiber bundles with no caliber variations along individual fibers have very small diffusivity time-dependence along the bundles at clinical diffusion time $t = 20 - 100$ ms, even for highly dispersed case ($\sim$ 0.5% diffusivity change for $\theta = 45°$). In contrast, realistic axonal shapes with caliber variations along each axon show significant diffusivity $1/\sqrt{t}$-dependence along axons ($\sim$ 5% diffusivity change for $\theta = 45°$ at $t = 20 - 100$ ms). To sum up, the diffusivity time-dependence along axons is mainly contributed by caliber variations along individual axons, instead of axonal diameter distribution across different axons.

Supplementary Tables

ROI	P-value	D_∞ (μm^2/ms)	c (μm^2·ms$^{-1/2}$)
ACR	6.4e-3	1.258 (0.019)	0.329 (0.105)
SCR	2.8e-4	1.328 (0.013)	0.413 (0.071)
PCR	1.3e-3	1.439 (0.013)	0.299 (0.074)
PLIC	4.0e-7	1.539 (0.006)	0.442 (0.033)
Genu	1.2e-2	1.484 (0.026)	0.389 (0.148)
Midbody	0.21	-	-
Splenium	8.9e-3	1.777 (0.018)	0.299 (0.103)
ALIC	1.2e-2	1.400 (0.017)	0.298 (0.101)

Supplementary Table 1. Fit parameters of the time-dependent axial diffusivity $D(t)$ in human brain data measured using monopolar PGSE (Supplementary Fig. 2a-b). Standard errors are shown in the parenthesis. (ACR/SCR/PCR = anterior/superior/posterior corona radiate, ALIC/PLIC = anterior/posterior limb of the internal capsule, genu/midbody/splenium of CC)

References

* Honghsi.Lee@nyulangone.org

[1] Katharine E. Stahon, Chinthasagar Bastian, Shelby Griffith, Grahame J. Kidd, Sylvain Brunet, and Selva Baltan. Age-related changes in axonal and mitochondrial ultrastructure and function in white matter. *The Journal of Neuroscience*, 36(39):9990–10001, 2016.

Supplementary Figures

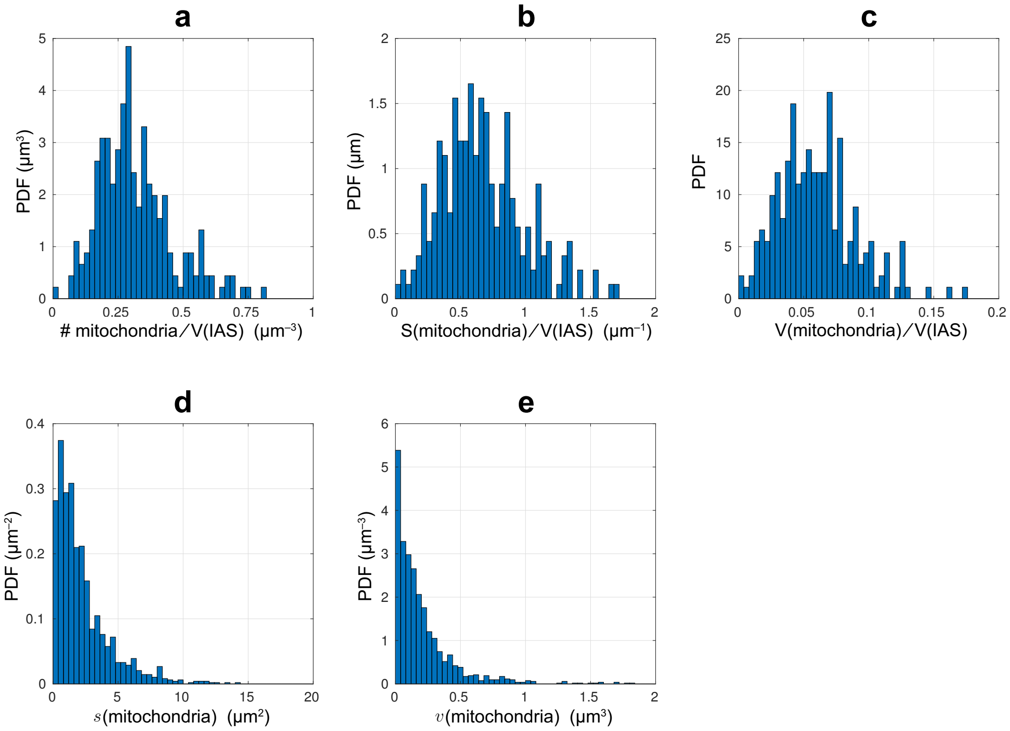

Supplementary Fig. 1. Mitochondrial morphometry based on the segmentation in Fig. 6a: a Histogram of the mitochondrial number per unit IAS volume for each axon. **b** Histogram of the ratio of mitochondrial surface area to IAS volume for each axon. **c** Histogram of the volume fraction of mitochondria to IAS for each axon. **d** Histogram of mitochondrial surface area of all segmented mitochondria. **e** Histogram of mitochondrial volume of all segmented mitochondria.

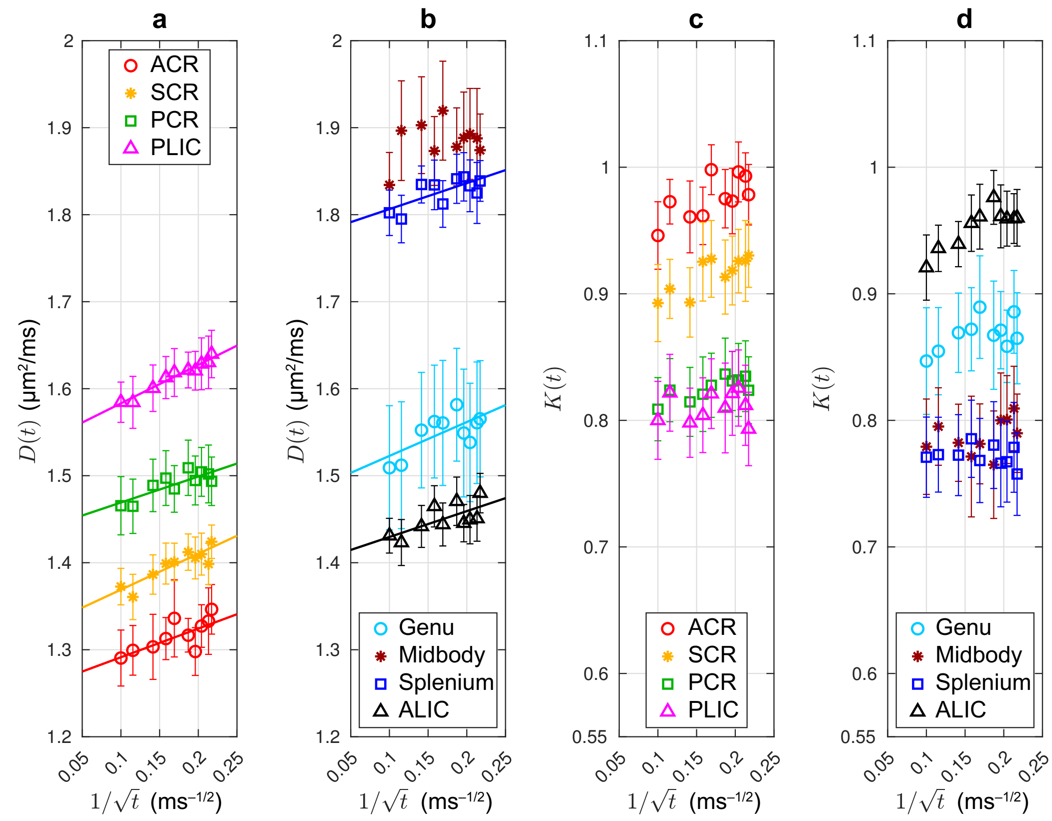

Supplementary Fig. 2. **a**, **b** Time-dependent axial diffusivity $D(t)$ measured in vivo in brain WM of 10 healthy subjects using monopolar PGSE. In all WM ROIs except Midbody of CC, the experimental axial diffusivity scales as $1/\sqrt{t}$ (P-value < 0.05, Supplementary Table 1), manifesting that the universality class along WM axons is short-range disorder in $1d$, corresponding to a power-law tail with $\vartheta = 1/2$. The fit parameters are summarized in Supplementary Table 1. **c**, **d** Time-dependent axial kurtosis $K(t)$ measured in vivo in brain WM of 10 healthy subjects using monopolar PGSE. The in vivo measured $K(t)$ is not zero, signifying the non-Gaussian diffusion along WM axons in the human brain. The error bar indicates the standard error of 10 subjects. (ACR/SCR/PCR = anterior/superior/posterior corona radiate, ALIC/PLIC = anterior/posterior limb of the internal capsule, genu/midbody/splenium of CC)

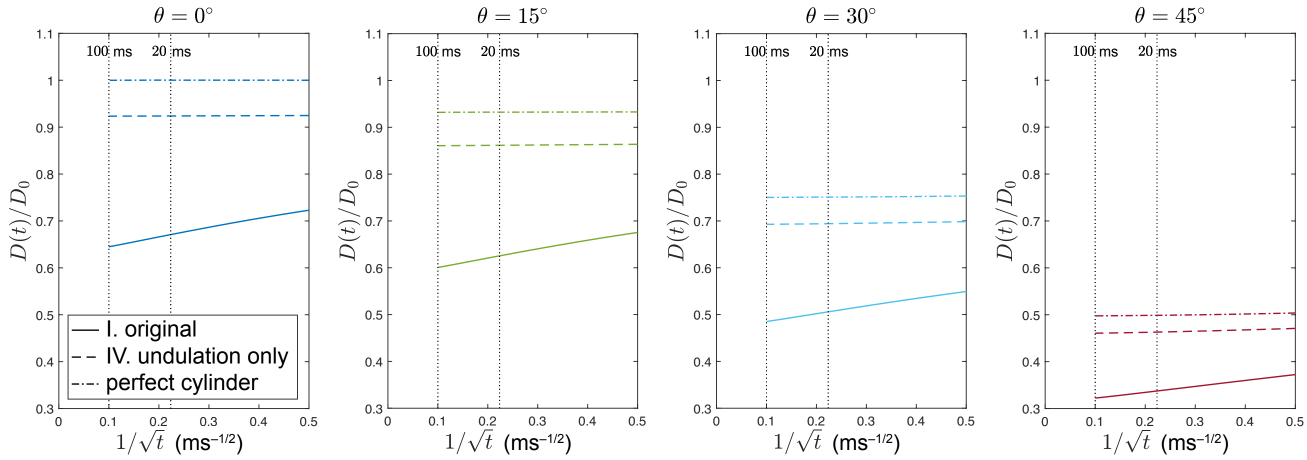

Supplementary Fig. 3. To demonstrate that the diffusivity time-dependence along axons is mainly contributed by caliber variations along individual axons, rather than diameter distribution across different axons, we performed Monte Carlo simulations in fiber bundles composed of (1) realistic IAS with caliber variations and axonal undulations (geometry I in Fig. 2a), (2) fibers with only axonal undulations (geometry IV in Fig. 2a), and (3) perfectly straight cylinders. The above three fiber bundles have the same $2d$ cross-sectional diameter distribution and orientation dispersion (Watson distribution). Simulation results show that fiber bundles with no caliber variations along individual fibers have negligible diffusivity time-dependence along the bundles at clinical diffusion time $t = 20 - 100$ ms, whereas realistic IAS has significant diffusivity $1/\sqrt{t}$-dependence along axons within the same time range.
