# Supplementary material for: A time-dependent diffusion MRI signature of axon caliber variations and beading
Source: Commun Biol. 2020 Jul 7;3:354. doi: 10.1038/s42003-020-1050-x (PMC7341838; doi:10.1038/s42003-020-1050-x)
Supplement: Supplementary file 2 — Description of Additional Supplementary Files [file 42003_2020_1050_MOESM2_ESM.pdf]

Description of Additional Supplementary Files

File Name: Supplementary Data 1
Description: All source data in main figures (Figs. 1-6) are available in the text format with a simple code for plotting.
