# Supplementary material for: A time-dependent diffusion MRI signature of axon caliber variations and beading
Source: Commun Biol. 2020 Jul 7;3:354. doi: 10.1038/s42003-020-1050-x (PMC7341838; doi:10.1038/s42003-020-1050-x)
Supplement: Supplementary file 4 — Reporting Summary [file 42003_2020_1050_MOESM4_ESM.pdf]

nature research

Corresponding author(s): Hong-Hsi Lee

Last updated by author(s): 5/27/2020

Reporting Summary

Nature Research wishes to improve the reproducibility of the work that we publish. This form provides structure for consistency and transparency in reporting. For further information on Nature Research policies, see Authors & Referees and the Editorial Policy Checklist .

Statistics

For all statistical analyses, confirm that the following items are present in the figure legend, table legend, main text, or Methods section.

n/a	Confirmed	
☐	☒	The exact sample size (*n*) for each experimental group/condition, given as a discrete number and unit of measurement
☐	☒	A statement on whether measurements were taken from distinct samples or whether the same sample was measured repeatedly
☐	☒	The statistical test(s) used AND whether they are one- or two-sided<br>*Only common tests should be described solely by name; describe more complex techniques in the Methods section.*
☐	☒	A description of all covariates tested
☐	☒	A description of any assumptions or corrections, such as tests of normality and adjustment for multiple comparisons
☐	☒	A full description of the statistical parameters including central tendency (e.g. means) or other basic estimates (e.g. regression coefficient) AND variation (e.g. standard deviation) or associated estimates of uncertainty (e.g. confidence intervals)
☐	☒	For null hypothesis testing, the test statistic (e.g. *F*, *t*, *r*) with confidence intervals, effect sizes, degrees of freedom and *P* value noted<br>*Give P values as exact values whenever suitable.*
☒	☐	For Bayesian analysis, information on the choice of priors and Markov chain Monte Carlo settings
☒	☐	For hierarchical and complex designs, identification of the appropriate level for tests and full reporting of outcomes
☐	☒	Estimates of effect sizes (e.g. Cohen's *d*, Pearson's *r*), indicating how they were calculated

Our web collection on statistics for biologists contains articles on many of the points above.

Software and code

Policy information about availability of computer code

Data collection	Monte Carlo simulations were performed by using Nvidia CUDA C++.<br><br>Brain tissue samples from a female 8-week-old C57BL/6 mouse's genu of corpus callosum were processed and analyzed with a scanning electron microscope (SEM) (Zeiss Gemini 300 SEM with 3View). To segment and reconstruct long axons passing through all slices, we employed a simplified seeded region growing algorithm based on (doi: 10.1007/s00429-019-01844-6) and (https://github.com/NYU-DiffusionMRI/RaW-seg).
Data analysis	Segmented axons were analyzed using the RaW-seg pipeline (https://github.com/NYU-DiffusionMRI/RaW-seg).<br><br>Monte Carlo simulation results were analyzed using MATLAB R2017a.<br><br>The following open-source software were used for analysis of MRI data:<br>1. Diffusion MRI processing DESIGNER pipeline, (doi: 10.1016/j.neuroimage.2018.07.066) and (https://github.com/NYU-DiffusionMRI/DESIGNER), which includes (1) Image denoising: dwidenoise in mrtrix3 (version 3), (2) Gibbs ringing correction: mrdegibbs in mrtrix3 (version 3), (3) motion and eddy current correction: FSL eddy (version 5.0.9), and (4) Rician bias correction (doi: 10.1016/j.jmr.2006.01.016).<br>2. Registration: FSL FLIRT and FNIRT (version 6.0.1)<br>3. Segmentation: FSL FAST (version 6.0.1)

For manuscripts utilizing custom algorithms or software that are central to the research but not yet described in published literature, software must be made available to editors/reviewers. We strongly encourage code deposition in a community repository (e.g. GitHub). See the Nature Research guidelines for submitting code & software for further information.

Data

Policy information about availability of data

All manuscripts must include a data availability statement. This statement should provide the following information, where applicable:

- Accession codes, unique identifiers, or web links for publicly available datasets
- A list of figures that have associated raw data
- A description of any restrictions on data availability

The SEM data and segmented axons can be downloaded on NYU CAI2R web page (http://cai2r.net/resources/software/intra-axonal-space-segmented-3d-scanning-electron-microscopy-mouse-brain-genu). All human brain MRI data for this study are available upon request.

Field-specific reporting

Please select the one below that is the best fit for your research. If you are not sure, read the appropriate sections before making your selection.

- [x] Life sciences
- [ ] Behavioural & social sciences
- [ ] Ecological, evolutionary & environmental sciences

For a reference copy of the document with all sections, see nature.com/documents/nr-reporting-summary-flat.pdf

Life sciences study design

All studies must disclose on these points even when the disclosure is negative.

Sample size	Five healthy subjects (and ten additional healthy subjects in Supplementary Information) are sufficient to estimate the scale and time-dependence of diffusivity in the normal brain white matter, as shown in the previous study (doi: 10.1016/j.neuroimage.2016.01.018). Similarly, we included five multiple sclerosis patients and compared diffusion metrics in lesions and normal appearing white matter.
Data exclusions	No data were excluded.
Replication	Two slightly different MRI protocols were applied on five and ten healthy subjects, respectively. The two protocols produced similar results and conclusions for diffusivity time-dependence in targeted white matter regions of interest.
Randomization	This is not relevant to this study since all samples and subjects are in the same group.
Blinding	This is not relevant to this study since all samples and subjects are in the same group.

Reporting for specific materials, systems and methods

We require information from authors about some types of materials, experimental systems and methods used in many studies. Here, indicate whether each material, system or method listed is relevant to your study. If you are not sure if a list item applies to your research, read the appropriate section before selecting a response.

Materials & experimental systems

n/a	Involved in the study	
x		Antibodies
x		Eukaryotic cell lines
x		Palaeontology
	x	Animals and other organisms
	x	Human research participants
x		Clinical data

Methods

n/a	Involved in the study	
x		ChIP-seq
x		Flow cytometry
	x	MRI-based neuroimaging

Animals and other organisms

Policy information about studies involving animals; ARRIVE guidelines recommended for reporting animal research

Laboratory animals	A female 8-week-old C57BL/6 mouse was perfused trans-cardiacally using a fixative solution of 4% PFA, 2.5% glutaraldehyde, and 0.1 M sucrose in 0.1 M phosphate buffer (PB, pH 7.4). The genu of corpus callosum was later excised from the midsagittal slice of the dissected brain, and the tissue was sampled from the central region of the genu and was fixed in the same fixative solution, followed by a PB containing 2% OsO4 and 1.5% potassium ferrocyanide for 1 h. The tissue was then stained with 1% thiocarbohydrazide (Electron Microscopy Sciences, EMS, PA) for 20 min, 2% osmium tetroxide for 30 min, and 1% aqueous uranyl acetate at 4 °C overnight. An En Bloc lead staining was performed at 60 °C for 30 min to enhance membrane contrast. The brain sample was dehydrated in alcohol and acetone, and embedded in Durcupan ACM resin (EMS, PA). The tissue sample was analyzed with a scanning electron microscope (Zeiss Gemini 300 SEM with 3View) at high-vacuum pressure, and 401 consecutive images of 6000 × 8000 pixels were acquired, representing a volume of 36×48×40.1 µm3 with a voxel size of 6×6×100 nm3. More

details are in (doi: 10.1007/s00429-019-01844-6).

Wild animals | The study did not involve wild animals.

Field-collected samples | The study did not involve samples collected from the field.

Ethics oversight | All procedures performed in this study involving animals were in accordance with the ethical standards of New York University School of Medicine. All mice were treated in strict accordance with guidelines outlined in the National Institutes of Health Guide for the Care and Use of Laboratory Animals, and the experimental procedures were performed in accordance with the Institutional Animal Care and Use Committee at the New York University School of Medicine.

Note that full information on the approval of the study protocol must also be provided in the manuscript.

Human research participants

Policy information about studies involving human research participants

Population characteristics | Five healthy subjects (4 males/1 female, 21-32 years old), ten additional healthy subjects (7 males/3 females, 23-30 years old) in Supplementary Information, and five multiple sclerosis patients (5 females, 32-48 years old)

Recruitment | We recruited subjects and patients who are between 18 and 99 years of age with documentation of MR-safe implant. Healthy subjects are employees, students and friends of staff at New York University School of Medicine. Patients have definite diagnosis of multiple sclerosis at New York University Langone Health.

Ethics oversight | All procedures performed in studies involving human participants were in accordance with the ethical standards of New York University School of Medicine. All protocols were approved by the local institutional review board (New York University School of Medicine). Informed consent was obtained from all individual participants included in the study.

Note that full information on the approval of the study protocol must also be provided in the manuscript.

Magnetic resonance imaging

Experimental design

Design type | Diffusion MRI with varying diffusion times, brain scan

Design specifications | Total scan time was 60 min for each subject.

Behavioral performance measures | Not applicable to regular diffusion time-dependent MRI scans.

Acquisition

Imaging type(s) | Structural MRI and Diffusion MRI

Field strength | 3 Tesla Siemens Prisma

Sequence & imaging parameters | 1. Five healthy subjects: Diffusion-weighted monopolar pulsed-gradient spin-echo sequence with EPI acquisition was used (Siemens WIP 919B), with an isotropic resolution 3x3x3 mm3 and a field-of-view 210x204 mm2 . The whole brain volume was scanned within 30 slices, aligned parallel to the anterior commissure-posterior commissure line. The matrix size was 70x68x30. GRAPPA with acceleration factor = 2 and multiband with acceleration factor = 2 were used. All scans were performed with the same TR/TE = 4000/139 ms.

2. Ten additional healthy subjects in Supplementary Information: Diffusion-weighted monopolar pulsed-gradient spin-echo sequence with EPI acquisition was used (Siemens WIP 511E) with an isotropic resolution 2x2x2 mm3 and a field-of-view 216x216 mm2 . The brain volume was scanned within 15 slices, aligned parallel to the anterior commissure-posterior commissure line. The matrix size was 108x108x15. GRAPPA with acceleration factor = 2 was used. All scans were performed with the same TR/TE = 5000/150 ms.

3. Five multiple sclerosis patients: Diffusion-weighted monopolar pulsed-gradient spin-echo sequence with EPI acquisition was used (Siemens WIP 511E) with an isotropic resolution 3x3x3 mm3 and a field-of-view 222x222 mm2 . The brain volume was scanned within 15 slices, aligned parallel to the anterior commissure-posterior commissure line. The matrix size was 74x74x15. GRAPPA with acceleration factor = 2 was used. All scans were performed with the same TR/TE = 4200/150 ms. Sagittal 3d MPRAGE brain images were acquired with an isotropic resolution 1x1x1 mm3, a field-of-view 256x256 mm2, TR/TE = 2100/2.72 ms, and inversion time = 900 ms. Axial FLAIR brain images were acquired with an anisotropic resolution 0.6875x0.6875x5 mm3, a field-of-view 220x220 mm2, TR/TE = 9000/90 ms, and inversion time = 2500 ms.

Area of acquisition | Whole brain scans were used for five healthy subjects. For ten additional healthy subjects and five multiple sclerosis patients, we scanned a slab of brain volume covering the major white matter regions of interests, such as corpus callosum.

Diffusion MRI ☒ Used ☐ Not used

Parameters	
	1. Five healthy subjects: 3 non-diffusion-weighted images and 62 diffusion-weighted images for three b-shells in total, with b-values = [400, 1000, 1500] s/mm2 along [12, 20, 30] directions respectively, diffusion time = 21.2-100 ms, diffusion gradient pulse width = 15 ms, no cardiac gating<br>2. Ten additional healthy subjects: 1 non-diffusion-weighted images and 64 diffusion-weighted images for four b-shells in total, with b-value = [100, 400, 1000, 1500] s/mm2 along [4, 10, 20, 30] directions respectively, diffusion time = 21.2-100 ms, diffusion gradient pulse width = 15 ms, no cardiac gating<br>3. Five multiple sclerosis patients: 3 non-diffusion-weighted images and 30 diffusion-weighted images for single b-shell, with b-value = 500 s/mm2, diffusion time = 21-110 ms, diffusion gradient pulse width = 15 ms, no cardiac gating

Preprocessing

Preprocessing software	Our image processing DESIGNER pipeline is based on (doi: 10.1016/j.neuroimage.2018.07.066) and (https://github.com/NYU-DiffusionMRI/DESIGNER).
Normalization	We transformed white matter regions of interest in John's Hopkins University DTI atlas to the individual subject space using linear and nonlinear transformation for quantitative analysis.
Normalization template	White matter regions of interest are transformed from John's Hopkins University DTI atlas to individual subject space using FSL FLIRT and FNIRT.
Noise and artifact removal	We removed the noise in diffusion weighted images using the MP-PCA algorithm (dwidenoise in mrtrix3) and eliminated the Gibbs ringing using mrdegibbs in mrtrix3. Motion and eddy current correction was performed by using FSL eddy.
Volume censoring	After motion and eddy current corrections, the top and the bottom slices were excluded.

Statistical modeling & inference

Model type and settings	Not applicable to regular diffusion time-dependent MRI scans.
Effect(s) tested	Not applicable to regular diffusion time-dependent MRI scans.

Specify type of analysis: ☐ Whole brain ☒ ROI-based ☐ Both

Anatomical location(s)	Major white matter regions of interest in John's Hopkins University DTI atlas were transformed to individual subject space using linear and nonlinear transformation.
Statistic type for inference (See Eklund et al. 2016)	Not applicable to regular diffusion time-dependent MRI scans.
Correction	Not applicable

Models & analysis

n/a	Involved in the study
☒	☐ Functional and/or effective connectivity
☒	☐ Graph analysis
☒	☐ Multivariate modeling or predictive analysis
